# Early Detection of Infants Aged Under Six Months at Risk of Poor Growth and Development: Secondary Analysis of a Prospective Cohort in Senegal

**DOI:** 10.1111/mcn.70275

**Published:** 2026-10-16

**Authors:** Tabitha D. van Immerzeel, Abou Ba, Fassiatou Tairrou, Amadou Diallo, Saliou Diouf, Babacar Faye, Elaine Ferguson, Princilia Mouissi, Sally Fall, Aicha Djigal, Ibrahima Diallo, Aliou A. Ndongo, Carlos S. Grijalva‐Eternod, Marko Kerac, Louise‐Tina Day, Hilary Davies‐Kershaw

**Affiliations:** ^1^ Department of Infectious Disease Epidemiology and International Health London School of Hygiene & Tropical Medicine London UK; ^2^ Institut de Pédiatrie Social University Cheick Anta Diop Dakar Senegal; ^3^ Department of Parasitology‐Mycology University Cheick Anta Diop Dakar Senegal; ^4^ Department of Epidemiology and Statistics Universiy Cheick Anta Diop Dakar Senegal; ^5^ Department of Epidemiology and Population Health London School of Hygiene & Tropical Medicine London UK

**Keywords:** growth and development, infant nutrition disorders, low birth weight, wasting

## Abstract

Child malnutrition is a major global problem, but in many high‐burden countries, screening and treatment of acute malnutrition called wasting (weight‐for‐length z‐score < −2SD) only begins at age 6 months. Infants under 6 months ‘at risk of poor growth and development’ are, however, increasingly recognised. Latest 2023 WHO guidelines describe anthropometric, feeding and maternal/infant clinical criteria for early detection and treatment. We aimed to explore the value of these criteria to early detect infants at risk of wasting at 6 months. We conducted a secondary analysis of a prospective observational cohort of 789 mother‐infant pairs in Senegal. Several WHO detection criteria were applied to examine the dyads and risk factors for wasting at 6 months, the current malnutrition programme enrolment criterion. Descriptive statistics, Alluvial plots and logistic regression analysis were employed. At birth, 6.8% of infants had low birth weight (weight < 2500 g) and most (99.7%) were breastfed. At age 3 months, 9.4% were underweight (weight‐for‐age z‐score < −2SD) and 10% were wasted; 66% were exclusively breastfed. Many infants transitioned between being malnourished and having normal weight over time. Wasting prevalence at 6 months was 9.5%; risk factors included low birth weight (adjusted odds ratio [aOR] 3.16, 95% CI:1.39, 7.19) and underweight at 3 months (aOR 6.62, 95% CI 2.96, 14.8). Infant feeding and maternal factors were not associated. Applying the 2023 WHO guidelines could detect infants at risk of poor growth and development early. Programme enrolment criteria need further refinement to identify infants requiring follow‐up or treatment.

## Key Messages

- Applying WHO anthropometric criteria, 31% of infants in our cohort were nutritionally at risk at least once during the study period, showing it is a common problem.
- Low birth weight and underweight at 3 months of age were associated with wasting at 6 months of age and could be detected early in routine clinical assessment.
- Since many infants transitioned between being nutritionally at risk and normal nutritional status during the first 6 months of life, observation and general support may be appropriate for many infants.

## 1 Introduction

Early detection and treatment of malnutrition are critical strategies in preventing child mortality globally (Victora et al. 2021). Wasting, defined as weight‐for‐length‐ z‐score (WLZ) < −2 SD, is often seen as a particularly high‐risk form of malnutrition, associated with increased risk of death (UNICEF 2025), and long‐term detrimental effects on child growth and neurodevelopment (Kirolos et al. 2022). Malnutrition prevention and treatment (therapeutic/supplementary feeding) programmes thus often target wasting.

Globally, 42.8 million (6.6%) children suffer from wasting, with the highest prevalences in Asia and Africa (UNICEF 2025). Community Management of Acute Malnutrition (CMAM) is a public‐health focused outpatient approach for management of wasting in children aged six to 59 months, aiming to reduce clinical case fatality (Collins et al. 2006). Since its introduction two decades ago, it has transformed the management of wasting with improved programme coverage, increased recovery and reduced mortality (Keats et al. 2021).

In contrast to CMAM success with older children, malnourished infants under 6 months of age (u6m) are often neglected and excluded from treatment programmes. This is partly due to the erroneous assumption that malnutrition is rare in this age group, for which exclusive breastfeeding is the target diet (Kerac et al. 2015). However, evidence shows that infants u6m do experience malnutrition, including wasting. Recent estimates indicate that 9.2 million (15.5%) infants u6m in low‐ and middle‐income countries are wasted (Kerac et al. 2025). Longitudinal data from 21 cohorts show that 29.2% of children experience at least one episode of wasting before 24 months of age (Mertens et al. 2023).

The need for improved detection and support for infants u6m has been widely expressed (van Immerzeel et al. 2022). To address the care gap, the 2023 updated WHO malnutrition guidelines introduced a section focussed on infants u6m emphasising early detection and outpatient treatment (WHO 2023). To ensure early and proactive identification of vulnerable infants u6m, the guidelines recognise a variety of risk factors and define infants u6m ‘at‐risk of poor growth and development’ as infants fulfilling one or more of the following criteria: (1) Poor birth outcomes (low birth weight, prematurity, small for gestational age), (2) Poor anthropometry based on a single measure: (WLZ < −2 SD, weight‐for‐age z‐score (WAZ) < −2 SD or mid‐upper‐arm‐circumference (MUAC) < 110 mm from 6 weeks onward), (3) Poor growth based on sequential measures, (4) Known risk factors for poor growth and development (maternal or infant‐related) (WHO 2023) (see Table A1 for the detailed list of criteria).

These WHO criteria are based on evidence synthesis, expertise in general child health/nutrition as well as programme experiences from the Managing small, nutritionally At‐ risk Infants u6m and their Mothers (MAMI) Care Pathway (Grey et al. 2021). The MAMI Care Pathway was developed by Emergency Nutrition Network (ENN) and uses similar enrolment criteria, with additional maternal criteria (Table A1).

The formal evidence base for many of the criteria to detect infants u6m at risk of poor growth and development remains limited (Daniel et al. 2025), making additional research necessary to corroborate and/or refine them. The present study aimed to contribute to strengthening the evidence by analysing a cohort of mother‐ infant dyads from Senegal. The objectives were:

– To describe clinical characteristics of infants u6m and their mothers,
– To examine anthropometric risk over time (at birth, 3 months and 6 months).
– To explore clinical risk factors for wasting at 6 months, based on a selection of the WHO criteria for ‘infants u6m at‐risk of poor growth and development’.

## 2 Methods

### 2.1 Study Design and Setting

This study is a secondary analysis of data from a prospective cohort study conducted by Action Against Stunting (AAS) Hub in Senegal, India and Indonesia. Detailed methods of this parent study have been published elsewhere (Davies‐Kershaw et al. 2024). For the present analysis, we used anthropometric and questionnaire data collected in Senegal from mothers and infants up to 6 months of age. We used Senegal data only to increase the clinical relevance of the study in current WHO guideline implementation, since some of the used criteria differ per context/country. Data were collected between March 2021 and June 2024 in Kaffrine, a rural area in central Senegal, where domestic farming is the primary source of income. The prevalence of wasting in children under five in Kaffrine is 9.3% (vs 10.2% nationally) (ANSD 2023). This study is reported following the STROBE guidelines (Cuschieri 2019).

### 2.2 Study Participants

Pregnant women in their first or second trimester were recruited during prenatal care visits. Exclusion criteria were age under 18 or over 40 years, residence outside the region and twin pregnancy. A total of 789 mother‐ infant pairs were enroled. There were five spontaneous abortions, 19 stillbirths and 15 infant deaths between birth and 6 months of age. At the time of the study, there were no specific nutritional intervention for infants u6m, but general advice was given during study visits. Due to (temporary) loss to follow‐up, there were 613 mother‐infant pairs at 3 months and 657 pairs at 6 months. The main reason for this low number at 3 months was the cultural habit of mothers moving to their family for an extended time after giving birth. Among other factors that affected rigorous data collection were distance between the study sites, limited phone connection and the use of community health workers as data collectors (Davies‐Kershaw et al. 2024).

### 2.3 Data Sources and Measurements

#### 2.3.1 Anthropometry

Anthropometric measurements were collected from pregnant women during the second and third trimester of pregnancy, and from infants within 72 h of birth, and at 3 and 6 months of age. Measurements were taken by trained midwives at health facilities. Weight was measured using the SECA‐876 scale with a mother‐child function (±0.1 kg precision). Maternal height and infant length were measured using a portable UNICEF height/length board (±0.1 cm precision). MUAC was measured using the Lufkin‐WP601 tape measure (±10 mm precision). Measurements were taken in duplicate, using standardised procedures and calibrated equipment, with a third measurement taken if the first and second measurements exceeded pre‐defined limits. A standardisation exercise took place prior to data collection, and regular refresher training was conducted. Field supervisors monitored data collection, using a checklist based on the standard operating procedures. Anthropometric z‐scores (WAZ and WLZ) were calculated using the 2006 WHO growth standards (WHO 2006).

#### 2.3.2 Demography, Feeding and Morbidity

A general questionnaire at enrolment included maternal occupation and education, water source and socio‐economic status (a composite variable including material goods, sanitation and living conditions). Within 72 h of birth, enumerators (public sector community health workers) administered a questionnaire on infant and young child feeding (IYCF) practices and health problems around the time of birth. Monthly follow‐up questionnaires on IYCF and morbidity were conducted face‐to‐face, either at the health facilities or participants' home.

#### 2.3.3 Maternal Mental Health

Signs of maternal depression were assessed using the Edinburgh Postnatal Depression Scale (EPDS) during the third trimester via face‐to‐face interviews conducted by the same enumerators in private settings (Ali et al. 2016).

### 2.4 Variable Selection

The primary outcome of interest was wasting at 6 months of age (defined as per WHO criteria WLZ < −2 SD), consistent with the main current CMAM malnutrition programme enrolment criteria. We treated wasting at 6 months as a proxy for the new definition ‘infants u6m at risk of poor growth and development’ (WHO 2023). MUAC and nutritional oedema as additional criteria for wasting or acute malnutrition were not included in the outcome variable because MUAC has a very limited diagnostic place in Senegal, ranking its usage lowest in a study comparing 47 countries (Grellety and Golden 2016), and nutritional oedema was not assessed in this cohort. However, we did a sensitivity analysis using WLZ < −2 or low MUAC (< 125 mm) as the outcome variable (Table A2). Predictor variables were selected based on the 2023 WHO criteria for infants u6m at‐risk of poor growth and development (WHO 2023) and the MAMI Care Pathway (Grey et al. 2021) (Table A1). Selection was further informed by the national health care plan (MSAS 2019) and clinical experience of the authors (TvI, AB, SD, LD), with the intention that this study's results could further orient WHO guideline implementation.

#### 2.4.1 Maternal Characteristics

Maternal age (defining < 19 years as adolescent as per MAMI Care Pathway), marital status, maternal education (no schooling, some primary and completed primary or higher), maternal occupation (paid vs unpaid work), water source (pipe‐borne vs other), socio economic status (three levels, only used for comparison analysis), maternal height and MUAC at second trimester (continuous and using MAMI Care Pathway cut‐offs: height < 145 cm, MUAC < 230 mm), maternal depression screening score (EPDS score < 13 vs > = 13).

#### 2.4.2 Pregnancy and Birth Characteristics

Previous live births (none vs one or more), antenatal care visits (0–3 vs 4 or more), delivery place (health facility vs home), delivery type (vaginal birth vs caesarean section), infants sex, weight and MUAC at birth, and the following infant binary characteristics: low birth weight (< 2500 g), low birth MUAC (using MAMI Care Pathway cut‐off < 110 mm), premature (< 37 weeks of gestation at birth, assessed by second trimester ultrasound (first choice) or last menstrual period (second choice)), ever breastfed, colostrum feeding, breastfeeding within the first hour of life, respiratory distress at birth.

#### 2.4.3 Infant Anthropometric, Feeding and Health at 3 and 6 Months

Weight, MUAC, underweight (WAZ < −2 SD), wasting (WLZ < −2 SD) or low MUAC (< 110 mm at 3 months, < 125 mm at 6 months). We created a composite indicator called ‘nutritionally at risk’, based on WHO criteria: low birth weight or premature at birth, underweight or wasting at 3 and 6 months. Other binary variables were exclusive breastfeeding (breastfeeding without any supplement), vaccination complete at 3 months, at least one illness episode reported between 1–3 and 4–6 months, at least one clinic visit reported between 1–3 and 4–6 months.

### 2.5 Data Analysis

To maximise the outputs and power, we used the entire sample available; we refer to the protocol paper of the parent study for a sample size calculation (Davies‐Kershaw et al. 2024). Descriptive statistics were calculated for all variables. For the descriptive analysis, we included those missing or lost to follow‐up as a separate group, since making this group of infants visible is clinically relevant in view of the implementation context of the updated WHO guidelines. We did a comparison analysis between those missing at 3 months and those not missing on factors such as sex, socio‐economic status, maternal factors and health‐related factors. Continuous variables were summarised as means and standard deviations, categorical variables as percentages. We calculated the prevalence of poor anthropometry defined by underweight, wasting and nutritionally at‐risk (defined above) at birth, 3 and 6 months as well as at one or a combination of these time points. Alluvial plots were used to visualise the anthropometric status over time, as described being useful in visualising longitudinal data (Brunson 2020).

Univariable logistic regression was used to assess associations and goodness of fit in a regression model between each predictor variable and wasting at 6 months, using the Wald test to calculate the crude odds ratio (OR). Predictor variables with *p*‐value of = < 0.20 in univariate analysis were then included in multivariate models (Shipe et al. 2019). We dealt with confounding in the following ways: we included sex in the models, which is a non‐modifiable variable correlated with nutrition (Thurstans et al. 2020). To adjust for time as a confounder, two distinct multivariable logistic regressions models were developed: Birth model based on variables collected during pregnancy or around birth, age 3–6 months model based on variables collected at between 3 and 6 months of age. To prevent collider bias of the different anthropometric measures, we selected one, most clinically relevant variable in the models, e.g. low birth weight at birth (Mwangome et al. 2019), underweight at 3 months (C. S. Grijalva‐Eternod et al. 2021). Whenever possible, we chose continuous over categorised variables, after having checked for departure from a linear trend. Since we aimed to look at exploring clinical risk factors in this population, instead of trying to understand causality (and to not overfit the models), we did not correct for other residual confounding. However, we do acknowledge that in maternal and child nutrition, many risk factors are interrelated and the causal diagram for the parent study visualises these complexities (Davies‐Kershaw et al. 2024). We applied the multiple imputation method to overcome the missing data of variables in the final models, using estimates from each imputed dataset combined to obtain pooled regression coefficients and standard errors (Austin and van Buuren 2022). The (adjusted) odds ratios and 95% confidence intervals were reported. All analyses were conducted using R version 4.2.2 (R Development Core Team 2010).

### 2.6 Ethical Considerations

Ethical approval for the parent study had previously been obtained from National Ethics Committee in Senegal (ref number SEN19/78) and from LSHTM (17915/RR/17513). Written informed consent was obtained from the infants' parents or legal guardians before enrolment. For our secondary analysis, we established a data sharing agreement with the parent study lead and obtained ethical approval from LSHTM (ref number 29833). We only used de‐identified participant data and data were shared using encrypted files.

## 3 Results

### 3.1 Description of the Cohort

Seven hundred and eighty‐nine mother–infant pairs were included in the study; 613 were assessed at 3 months and 657 at 6 months. Comparison analysis between those lost and those who remained at 3 months did not show important differences (Table A3). Mothers' mean age at enrolment was 25.4 (SD 5.8) years. Nearly half had no formal education, and most were engaged in unpaid domestic work (Table 1). Maternal short stature was rare (three mothers), while 17% had a MUAC < 230 mm. The majority had attended four or more antenatal care visits; caesarean section deliveries accounted for 5.8%, and home delivery was uncommon. 14% of mothers showed signs of maternal depression.

**Table 1:** Maternal and infant characteristics of the study participants at pregnancy/birth, 3 months and 6 months of age.

			Total sample: *N* = 789	
Variable	Category	*N*	Mean (SD)	*n* (%)
Maternal characteristics				
Maternal age, years		766	25.4 (5.8)	
Maternal age	< 19 years			67 (8.7)
Marital status	Married	757		727 (96)
Maternal education	No schooling	687		319 (46)
	Some primary			266 (39)
	Primary or higher			102 (15)
Maternal occupation	Housewife/unpaid work	766		645 (84)
	Paid work			121 (16)
Water source	Pipe‐borne	748		662 (89)
	Other			86 (11)
Maternal height, cm		747	164.9 (6.4)	
Maternal height	< 145 cm			3 (0.4)
Maternal MUAC, mm		743	259 (35)	
Maternal MUAC	< 230 mm			129 (17)
EPDS score		748	6.3 (5.1)	
Maternal depression	EPDS score ≥ 13			108 (14)
Pregnancy and birth characteristics				
Previous livebirths	None	599		33 (5.5)
	One or more			566 (94)
Antenatal care visits	0–3	745		274 (37)
	4 or more			471 (63)
Delivery place	Home	776		14 (1.8)
	Health facility			762 (98)
Delivery type	Caesarean section	756		44 (5.8)
Infant sex	Male	766		367 (48)
Birth weight, g		774	314 (0.5.1)	
Low birth weight (< 2500 g)	Yes			53 (6.8)
Birth MUAC, mm		736	108.8 (10.6)	
Low birth MUAC	< 110 mm			242 (33)
Gestational age at birth (weeks)		743	39.0 (2.1)	
Prematurity (GA at birth < 37w)	Yes			83 (11)
Ever breastfed	Yes	738		736 (99.7)
Initiation of breastfeeding	< 1 h	740		496 (67)
Colostrum feeding	Yes	732		693 (95)
Respiratory distress at birth	Yes	732		50 (6.8)
Antibiotic at birth	Yes	735		373 (51)
Infant anthropometric and health at 3 and 6 months of age				
Weight at 3 m, g		612	594 (8.5)	
MUAC at 3 m, mm		613	129.5 (11.2)	
Low MUAC at 3 m	< 110 mm			16 (2.6)
WAZ at 3 m		609	−0.54 (1.13)	
Underweight at 3 m	WAZ < −2SD			57 (9.4)
WLZ at 3 m		601	−0.51 (1.25)	
Wasting at 3 m	WLZ < −2SD			63 (10)
Exclusive breastfeeding at 3 m	Yes	505		330 (66)
Immunisation complete at 3 m	Yes	600		414 (69)
Illness episode § 1–3 m	1 or more	743		119 (16)
Sick child consultation 1–3 m	1 or more	679		71 (10)
Weight at 6 m, g			718 (100)	
MUAC at 6 m, mm		653	135.9 (11.2)	
Low MUAC at 6 m	< 125 mm			90 (14)
WAZ at 6 m		649	−0.71 (1.15)	
Underweight at 6 m	WAZ < −2 SD			89 (14)
Severe underweight at 6 m	WAZ < −3 SD			13 (2.0)
WLZ at 6 m		643	−0.56 (1.12)	
Wasting at 6 m	WLZ < −2 SD			61 (9.5)
Severe wasting at 6 m	WLZ < −3 SD			11 (1.7)
Exclusive breastfeeding at 6 m	Yes	387		186 (48)
Illness episode‡ 4–6 m	1 or more	678		142 (21)
Sick child consultation 4–6 m	1 or more	678		104 (15)

Abbreviations: EPDS = Edinburgh Postnatal Depression Scale, GA = gestational age, MUAC = mid‐upper arm circumference, WAZ = weight‐for‐age z‐score, WLZ = weight‐for‐length z‐score.

^‡^ Defined as at least one illness episode reported between 1–3/4–6 months.

Among infants, 53 (6.8%) had low birth weight and 83 (11%) were born premature. Nearly all mothers initiated breastfeeding, although one third did not begin within the first hour. Exclusive breastfeeding declined from 99.7% at birth to 66% at 3 months and 48% at 6 months. Two‐thirds of infants were fully vaccinated by 3 months. Recent illness episodes were reported in 16% of infants at 3 months and 21% at 6 months of age.

### 3.2 Anthropometric Deficits at the Three Time Points

Table 2 presents the prevalence of three malnutrition indicators: underweight, wasting and ‘nutritionally at risk’ at birth, 3 months and 6 months. Our outcome variable wasting at 6 months was observed in 61 infants (9.5%). One third of infants (31%) were nutritionally at risk at least once, and few (3.8%) at all three time points. Wasting prevalence was highest at birth (20%) and declined over time (10% at 3 months, 9.5% at 6 months), while underweight prevalence increased from 6% at birth to 14% at 6 months.

**Table 2:** Prevalence of underweight, wasting and ‘nutritionally at risk’ at birth, 3, and 6 months and combinations of timepoints.

	Prevalence at timepoint			At least at one timepoint	Uniquely at one timepoint			At 2 timepoints			At 3 timepoints
	*N* = 783	*N* = 613	*N* = 657	*N* = 783	*N* = 783	*N* = 613	*N* = 657	*N* = 613	*N* = 657	*N* = 613	*N* = 613
	Birth	3 months	6 months	Birth or 3 m or 6 m	Birth only	3 m only	6 m only	Birth & 3 m	Birth & 6 m	3 m & 6 m	Birth & 3 m & 6 m
Underweight (WAZ < −2SD)	44 (6.0%)	57 (9.4%)	89 (14%)	136 (18%)	24 (3.3%)	16 (2.8%)	45 (7.2%)	12 (2.1%)	17 (2.7%)	34 (6.4%)	9 (1.8%)
Missing	52	4	8	22	45	32	34	28	33	82	101
Wasting (WLZ < −2SD)	141 (20%)	63 (10%)	61 (9.5%)	222 (29%)	114 (16%)	23 (4.3%)	32 (5.6%)	17 (3.1%)	16 (2.7%)	18 (3.5%)	8 (1.7%)
Missing	89	12	14	20	109	77	81	68	73	141	143
Nutritionally at risk†	121 (16%)	93 (16%)	107 (17%)	238 (31%)	79 (11%)	35 (6.1%)	47 (7.7%)	26 (4.5%)	32 (5.2%)	44 (8.5%)	19 (3.8%)
Missing	45	19	14	18	67	43	44	34	37	140	112

Abbreviations: WAZ = weight‐for‐age z‐score, WLZ = weight‐for‐length z‐score.

^†^ At birth: low birth weight or premature; at 3 months or 6 months: underweight or wasting.

Figure 1 (Alluvial plots) illustrates the infants being wasted, underweight or nutritionally at risk at 6 months and their nutritional status at birth and 3 months of age, showing a variety of trajectories of infants transitioning between being malnourished and having a normal nutritional status. Many of those being malnourished at 6 months had already been malnourished at 3 months and/or at birth. Most missing data were observed at the 3‐month time point.

**Figure 1:**
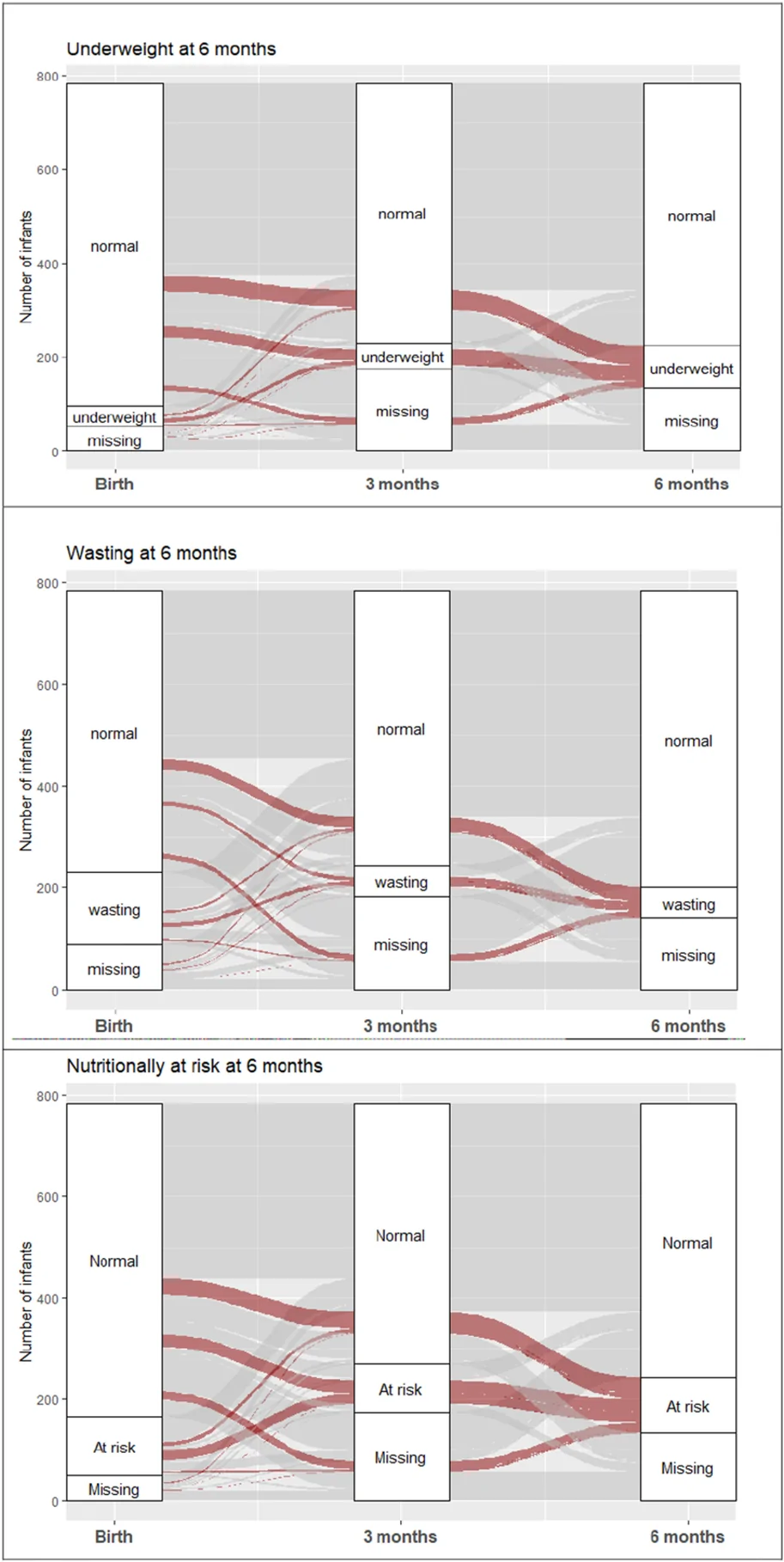
Alluvial plot of underweight, wasting and nutritionally at risk at 6 months and their nutritional status at birth and 3 months. Definitions: underweight = WAZ < −2 SD (red), not underweight or missing (grey), wasting WLZ < −2 SD (red), not wasted or missing (grey), nutritionally at‐risk at birth = low birth weight or premature at birth; wasting or underweight at 3 or 6 months (red), not nutritionally at risk or missing (grey), missing = includes both the (temporary) loss to follow‐up and the missing values.

### 3.3 Risk Factors for Wasting

Univariable logistic regression analysis identified low birth weight as associated with wasting at 6 months of age, as well as infants receiving an antibiotic at birth. All the anthropometric indicators (underweight, wasting, low MUAC) at 3 months were individually significantly (*p*‐value < 0.05) associated with wasting at 6 months of age. Feeding related factors were not significantly associated (Table 3).

**Table 3:** Results of univariable logistic regression analysis with wasting (WLZ < −2 SD) at 6 months of age as outcome (vs non‐wasted).

Variables	N	OR	95% CI	*p*‐value
Maternal factors				
Maternal age (years)	643	1.04	1.00, 1.09	0.076
Maternal height (cm)	627	1.02	0.98, 1.07	0.4
Maternal MUAC (mm) (second trimester)	625	0.92	0.84, 1.00	0.055
Maternal MUAC < 230 mm (second trimester)	625	1.58	0.81, 2.93	0.2
Antenatal care: 0–3 visits	625	1.34	0.78, 2.30	0.3
Previous liveborn: none	503	1.34	0.78, 2.30	0.3
Type of delivery: c‐section	634	0.80	0.19, 2.30	0.7
EPDS depression score (third trimester)	628	0.99	0.93, 1.04	0.6
Poor birth outcomes				
Birth weight (g)	637	0.65	0.38, 1.10	0.11
Low birth weight (< 2500 g)	637	2.68	1.16, 5.66	0.014
Gestational age (weeks)	625	1.12	0.97, 1.29	0.13
Premature (born < 37weeks GA)	625	0.53	0.16, 1.35	0.2
MUAC at birth (mm)	611	0.85	0.66, 1.09	0.2
MUAC at birth < 110 mm	611	1.17	0.66, 2.01	0.6
Poor anthropometry based on single measure				
Weight at 3 months (g)	525	0.27	0.17, 0.40	< 0.001
WAZ at 3 months	524	0.38	0.28, 0.50	< 0.001
Underweight at 3 months (WAZ < −2 SD)	524	7.69	3.74, 15.5	< 0.001
WLZ at 3 months	516	0.36	0.26, 0.48	< 0.001
Wasting at 3 months (WLZ < −02 SD)	516	11.0	5.38, 22.5	< 0.001
MUAC at 3 months (mm)	526	0.36	0.25, 0.49	< 0.001
Low MUAC (< 110 mm) at 3 months	526	5.34	2.50, 11.0	< 0.001
Other clinical risk factors				
Breastfeeding not within 1 h	622	1.17	0.66, 2.02	0.6
Breastfeeding no initiation	619	—	—	> 0.9
Respiratory distress at birth	614	0.24	0.01, 1.16	0.2
Antibiotic at birth	607	1.76	1.02, 3.10	0.046
Non‐exclusive breastfeeding at 3 months	432	1.01	0.50, 1.95	> 0.9
Not breastfeeding at 6 months	542	—	—	> 0.9
Immunisation incomplete at 3 months	548	1.86	0.81, 3.88	0.12
At least one illness episode 1–3 months	616	0.64	0.26, 1.36	0.3
At least one clinic consultation 1–3 months	574	0.86	0.29, 2.07	0.8
At least one illness episode 4–6 months	588	1.52	0.80, 2.76	0.2
At least one clinic consultation 4–6 months	588	1.38	0.66, 2.69	0.4

*Note:* All variables are binary, except those expressed in measurement units.

Abbreviations: GA = gestational age, MUAC = mid‐upper arm circumference, WAZ = weight‐for‐age z‐score, WLZ = weight‐for‐length z‐score.

In the multivariable logistic regression birth model, low birth weight remained a risk factor (aOR 3.16, 95% CI 1.39, 7.19), along with infant receiving an antibiotic at birth (aOR 1.91, 95% CI 1.09, 3.38). In the 3–6 months model, underweight at 3 months was selected among anthropometric indicators (explained in the methods section) and remained significantly associated with wasting at 6 months (aOR 6.62, 95%CI 2.96, 14.8) (Table 4).

**Table 4:** Multivariable logistic regression analysis: birth model and age 3–6 months model with wasting (WLZ < −2 SD) at 6 months of age as outcome (vs non‐wasted).

	Birth model			
Characteristic	Type	aOR	95% CI	*p*‐value
Low birth weight	Binary	3.16	1.39, 7.19	0.006*
Infant received antibiotic at birth	Binary	1.91	1.09, 3.38	0.024*
Maternal MUAC	Continuous	0.94	0.86, 1.02	0.15
Male sex	Binary	1.40	0.81, 2.42	0.22

	Age 3–6 months model			
Characteristic	Type	aOR	95% CI	*p*‐value
Underweight (WAZ < −2) at 3 months	Binary	6.62	2.96, 14.80	< 0.001*
Incomplete immunisation at 3 months	Binary	1.98	0.86, 4.55	0.11
Recent illness episode (4–6 months)	Binary	1.58	0.83, 3.08	0.16
Male sex	Binary	1.30	0.72, 2.33	0.38

Abbreviations: aOR = adjusted Odds ratio, CI = confidence interval, MUAC = mid‐upper arm circumference, WAZ = weight‐for‐age z‐score.

* *p* value < 0.05.

## 4 Discussion

In this cohort of mothers and infants in Senegal, several WHO detection criteria for infants at risk of poor growth and development could be identified between birth and 6 months, such as low birth weight (6.8%), nonexclusive breastfeeding (34% at 3 months), signs of maternal depression (14%), and infant anthropometric deficits. Using the anthropometric criteria, 31% of infants were at risk at least once during the first 6 months of life. Despite an absence of direct treatment, many transitioned between malnutrition and normal status over this time. In this cohort, we found that low birth weight and underweight at age 3 months were statistically significant risk factors for later wasting, at age 6 months.

### 4.1 Maternal Factors

Maternal education levels (46% with no schooling) were comparable to rural DHS estimates (51%) (ANSD 2023). Due to the study's exclusion criteria, adolescent mothers were underrepresented in this cohort and could therefore not be fully assessed as a risk factor. In Senegal, 18.3% of 18‐year‐old women have already had a pregnancy (ANSD 2023), making this an important potential risk factor for infant malnutrition (UNICEF 2023). 14% of the mothers had a high mental health score, but having a high score was not correlated with infant wasting at 6 months. This corresponds with findings from a cross‐sectional study in Ethiopia (Abera et al. 2024). A systematic review in LMICs, however, did show higher odds for malnutrition if mothers suffered postnatal depression (Carosella et al. 2024). There were no significant associations of maternal factors with wasting in this cohort. However, when low MUAC was added to the outcome variable at 6 months, lower maternal MUAC during pregnancy was associated with the outcome (see Table A2). Low maternal MUAC has been associated with child malnutrition in a cohort in Bangladesh (Munirul Islam et al. 2019) as well as in a large cross‐sectional study among South Sudanese refugees (Moore et al. 2021).

### 4.2 Birth Factors

The low‐birth‐weight prevalence (6.8%) was lower than DHS estimates (15%), possibly due to differences in methods (written vs orally reported birth weight). Prematurity was 11% in our study, though gestational age assessment via ultrasound was limited, which may have affected classification. This lack of assessment is a common cause of underreporting of prematurity and small for gestational age (Lawn et al. 2023). Low birth weight was strongly correlated with wasting at 6 months in our study. Adverse birth outcomes are well‐known risk factors for later malnutrition: Globally, more than one in four children are born small and vulnerable, contributing to over half of neonatal deaths (Lawn et al. 2023). Birth weight is not always documented in clinical settings, and accuracy is an issue in registration with common problems like heaping (Kong et al. 2021). Small for gestational age is an even better predictor for later malnutrition (Nel et al. 2024), but this could not be calculated in our dataset. Low MUAC at birth was shown to be well correlated with wasting under 6 months of age in India (Seth et al. 2021), but our study did not confirm this.

Receiving an antibiotic at birth was associated with the outcome. Half of all infants received an antibiotic at birth, which can be explained by local guidelines recommending low‐threshold antibiotic prescription at birth (including for long (> 12 h) or short (< 6 h) duration of labour, or difficult labour) (MSAS 2020). Since indications include preventative treatment, this variable appears to be a weak proxy for infant illness at birth, and the association with wasting must be interpreted considering this. Caesarean section rates were slightly lower than national averages (5.8% vs 9.3%), possibly reflecting the rural setting. Overall, 6.8% of infants suffered from respiratory distress at birth, which corresponds to estimates in LMIC (Sivanandan et al. 2017).

### 4.3 Infant Anthropometry

The prevalence of wasting at 6 months in our cohort (9.5%) aligns with recent 2023 DHS data for Senegal (9.7% in infants aged 6–11 months) (ANSD 2023). It is, however, lower than the 14.6% average reported across 56‐country DHS analyses for infants 0–6 months (Kerac et al. 2025). Trends in anthropometric indicators at the three time points varied: Underweight prevalence increased over time, while wasting decreased. This is consistent with findings from a synthesis of 21 longitudinal studies in LMIC (Mertens et al. 2023). A possible explanation provided by the latter study is that, compared to underweight, wasting at birth is more related to pregnancy‐related factors such as placental insufficiency, with a rapid catch‐up weight gain after birth.

Our longitudinal findings add to this knowledge that individual infants follow different trajectories of nutritional status (Figure 1). Many infants who were malnourished at 6 months of age were malnourished at age 3 months, and could have been detected early. However, almost one‐third (31%) of all infants were nutritionally at risk at least once during the study period, which potentially has important programmatic implications (Negesse et al. 2025). It might be necessary to prioritise anthropometric measures to reduce caseload. WAZ showed the most overlap with other anthropometric deficits in a cross‐sectional study on health service level in Ethiopia, thus capturing malnutrition u6m well (Carlos S Grijalva‐Eternod et al. 2021). Moreover, the recent WHO Risk Stratification Working Group (WHO‐RSWG) pooled data from 33 studies across 17 countries and found that underweight was a better predictor of mortality than wasting in this age group (Ahmed et al. 2025).

### 4.4 Feeding and Health

Exclusive breastfeeding rates were higher than national averages (66% at 3 months and 48% at 6 months, vs. 37.9% and 13.9%). This discrepancy is likely influenced by social desirability bias: enumerators in our study were well‐known community health workers, providing regular IYCF counselling. Breastfeeding indicators are known to be overreported for various reasons when comparing with direct observation (Day et al. 2021). In reality, exclusive breastfeeding rates have remained low over the past decade in Senegal (Camara et al. 2024). Unlike some other studies, we found no association between feeding practices and later wasting, possibly because of this bias. In a cross‐sectional study in Ethiopia, infants were 50% more likely to be wasted at 6m when not exclusively breastfed or when breastfeeding was not initiated early (Getachew et al. 2025). In Bangladesh, both mothers' low satisfaction with breastfeeding as well as a low frequency of breastfeeding were correlated with wasting at 6 months of age (Munirul Islam et al. 2019).

Our morbidity variable based on reported illness at 6 months (21%) corresponds to DHS data (24%) (ANSD 2023). The presence of illness episodes or clinic visits was not associated with wasting in our study. Some earlier studies reported an association between acute illness like diarrhoea and wasting (Fekadu et al. 2015), while others did not (Choudhary et al. 2019; Munirul Islam et al. 2019). We know that the effect between illness and malnutrition can be bidirectional (Karakochuk et al. 2018), and the WHO guideline recommendation to conduct a clinical assessment for all at‐risk infants u6m is therefore relevant.

### 4.5 Strengths and Limitations

Strengths of this study include the longitudinal design, allowing to follow risk factors over time, and rigorous quality control of anthropometric measurements. We also directly inform a topic where evidence is urgently needed (Kerac et al. 2025). We do, however, acknowledge limitations. One is that our outcome variable wasting does not capture the full WHO definition of ‘poor growth and development’, nor final mortality outcomes. Longer‐term outcome data on development milestones and later mortality/morbidity would provide the possibility for more robust risk factors analysis or developing a clinical prediction score.

Also, not all variables of interest (e.g. detection criteria of the 2023 WHO guidelines) were collected, such as multiple births and adolescent mothers, which were exclusion criteria for the study. This limits the generalisability of the results and applicability in WHO guideline implementation. There was a risk of selection bias: study participants were selected during prenatal care, not selecting those who came late or not at all for prenatal visits. The recall bias in collecting feeding and morbidity data is a limitation, but reflects the reality in a clinical setting, where health workers often rely on recall data. The large number of loss to follow‐up could have induced attrition bias. However, the comparison analysis between those lost to follow‐up at 3 months and those who remained (see Table A3) did not show differences in baseline characteristics.

### 4.6 Implications

The early start of malnutrition in this cohort underlines the importance of the WHO malnutrition guidelines for infants u6m. In Senegal, infants u6m and their mothers already engage well with the primary healthcare system and outpatient services (e.g. postnatal care and immunisation services), providing opportunities for early detection and management of those at risk of later wasting (van Immerzeel et al. 2025). For example, early and regular weight monitoring could identify at‐risk infants. Weight is easier to measure accurately than length and is already part of routine care at multiple contact points in primary care (Hoehn et al. 2021). Given the transitions in nutritional status during the first 6 months of life, some infants classified as at‐risk might be safely just observed rather than needing treatment. Besides anthropometric criteria, infant and maternal health factors need to be considered in the case definition. Instead of simple anthropometric criteria for enrolment and follow‐up as with older children, infants u6m need a more individual, risk‐based approach (Berkley et al. 2024).

## 5 Conclusion

This cohort study of mothers and infants in Senegal showed low birth weight and underweight at 3 months to be early risk factors for wasting at 6 months of age. Using WHO anthropometric criteria, 31% of infants in our cohort were nutritionally at risk at least once during the study period. Given the many transitions in nutritional status during the first 6 months of life, early and subsequent weight measurement will be crucial in detecting those who need follow‐up or care.

## Author Contributions

TvI, L.T.D., M.K., C.G. and H.D. designed the study. B.F., E.F., S.D., A.B. and H.D. were overall responsible for the parent study. TvI, A.N., P.M., S.F., I.D. and Ai.D. supervised different parts of the data collection. H.D., T.vI and F.T. cleaned the data. TvI did the principal analysis. H.D., F.T. and Am.D. contributed to the analysis and visualisation. T.vI wrote the manuscript, and all authors reviewed several drafts. All authors have approved the final manuscript.

## Conflicts of Interest

The authors declare no conflicts of interest.

## Data Availability

Data from Action Against Stunting Hub Senegal were used under license. Due to third‐party terms, these data cannot be shared openly but are available upon reasonable request to the authors for research purposes, subject to data use agreements.

## Appendix 1

Table A1

**Table A1:** WHO criteria for infants u6m at risk of poor growth and development, with MAMI Care Pathway (MAMI‐CP) criteria.

WHO category	Type	Definition	WHO	MAMI‐CP
Poor birth outcomes	Low birth weight	Weight at birth < 2500 grams	x	x
	Premature	Born < 37 weeks of pregnancy	x	x
	Small for gestational age	Infants below the 10th centile of a birthweight‐for‐gestational‐age reference	x	x
	Multiple birth			x
Poor anthropometry u6m	Underweight	Weight‐for‐age z‐score (WAZ) < −2	x	x
	Wasting	Weight‐for‐length z‐score (WLZ) < −2	x	x
		MUAC < 110 at birth		x
		MUAC < 110 mm 6 weeks to 6 months	x	x
Poor growth u6m	Growth failure	Insufficient weight gain or weight loss from one measurement to the next	x	
		Downward crossing of weight‐for‐age centile	x	
		Failure to gain a minimum of 150 g/week 0–3 months or 100 g/week 4–6 months		x
Other risk factors	Neurodevelopmental concerns		x	x
	Infant feeding or health concerns	Infant not breastfed		x
		Infant receives other food/drinks		x
		Infant illness that effects feeding	x	x
		Physical or mental health problem affecting caring practices	x	x
	Maternal risk	Adolescent mother (age < 19 years)		x
		Maternal height < 145 cm		x
		Maternal MUAC < 230 mm		x
		Maternal high depression score		x

## Appendix 2

Table A2

**Table A2:** Multivariable logistic regression analysis: birth model and age 3–6 months model with acute malnutrition (WLZ < −2 SD or MUAC < 125 mm) at 6 months of age as outcome (vs no acute malnutrition).

	Birth model			
Characteristic	Type	aOR	95% CI	*p*‐value
Low birth weight	Binary	3.58	1.82, 7.03	< 0.001*
Infant received antibiotic at birth	Binary	1.14	0.75, 1.74	0.54
Maternal MUAC	Continuous	0.91	0.85, 0.98	0.013*
Male sex	Binary	0.61	0.40, 0.94	0.025*

	Age 3–6 months model			
Characteristic	Type	aOR1	95% CI	*p*‐value
Underweight (WAZ < −2) at 3 months	Binary	6.43	2.79, 14.8	< 0.001*
Incomplete immunisation at 3 months	Binary	1.30	0.63, 2.68	0.48
Recent illness episode (4–6 months)	Binary	1.50	0.88, 2.53	0.13
Male sex	Binary	0.60	0.37, 0.97	0.037*

Abbreviations: aOR = adjusted Odds ratio, CI = confidence interval, MUAC = mid‐upper arm circumference, WAZ = weight‐for‐age z‐score.

* *p*‐value < 0.05.

## Appendix 3

Table A3

**Table A3:** Comparison analysis between infants missing at 3 months vs those non‐missing, on basic characteristics.

	Missing at 3 months	Non‐missing at 3 months
Comparison analysis	*N* = 177	*N* = 612
Maternal age, years (mean, SD)	25.3 (5.9)	25.4 (5.8)
Marital status, married	143/152 (94%)	584/605 (97%)
Maternal education		
completed primary or higher	27/142 (19%)	75/545 (14%)
No schooling	63/142 (44%)	256/545 (47%)
Some primary	52/142 (37%)	214/545 (39%)
Maternal occupation, housewife	122/154 (79%)	523/612 (85%)
Socioeconomic status		
Highest wealth	50/170 (29%)	209/612 (34%)
Lowest wealth	60/170 (35%)	212/612 (35%)
Mid‐wealth	60/170 (35%)	191/612 (31%)
Water source, pipe‐borne	131/152 (86%)	531/596 (89%)
Maternal MUAC (mm)	257 (3.3)	259 (3.5)
Antenatal care visits, 4 or more	104/152 (68%)	367/593 (62%)
EPDS score	6.1 (4.9)	6.4 (5.2)
Male sex	82/154 (53%)	285/612 (47%)
Birth weight	3.15 (0.53)	3.14 (0.50)
Low birth weight	10/167 (6.0%)	43/607 (7.1%)
Prematurity	21/152 (14%)	62/591 (10%)
Breastfeeding within 1 h	96/150 (64%)	400/590 (68%)
Respiratory distress at birth	10/147 (6.8%)	40/585 (6.8%)
Wasting at 6 months	14/118 (12%)	47/525 (9.0%)
